# Backbone N-Amination Promotes the Folding of β-Hairpin Peptides via a Network of Hydrogen Bonds

**DOI:** 10.1021/acs.jcim.2c00516

**Published:** 2022-07-11

**Authors:** Jožica Dolenc, Esme J. Haywood, Tingting Zhu, Lorna J. Smith

**Affiliations:** † Chemistry | Biology | Pharmacy Information Center, ETH Zurich, Zurich CH-8093, Switzerland; ‡ Inorganic Chemistry Laboratory, Department of Chemistry, University of Oxford, South Parks Road, Oxford OX1 3QR, United Kingdom

## Abstract

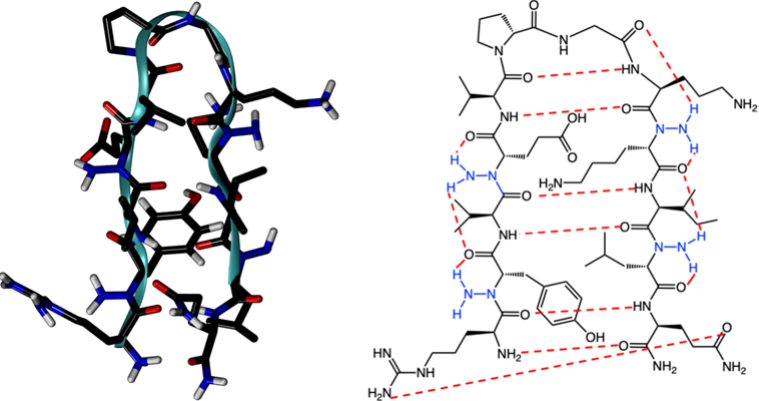

Molecular dynamics (MD) simulations have been used to characterize the effects of backbone N-amination of residues in a model β-hairpin peptide. This modification is of considerable interest as N-aminated peptides have been shown to inhibit amyloid-type aggregation. Six derivatives of the β-hairpin peptide, which contain one, two, or four N-aminated residues, have been studied. For each peptide 100 ns MD simulations starting from the folded β-hairpin structure were performed. The effects of the N-amination prove to be very sequence dependent. N-Amination of a residue involved in interstrand hydrogen bonding (Val3) leads to unfolding of the β-hairpin, whereas N-amination of a residue toward the C-terminus (Leu11) gives fraying at the termini of the peptide. In the other derivatives the peptide remains folded, with increasing levels of N-amination reducing the right-handed twist of the β-hairpin and favoring population of a type II′ rather than a type I′ β-turn. MD simulations (100 ns) have also been run for each peptide starting from an unfolded extended chain. Here, the peptide with four N-aminated residues shows the most folding into the β-hairpin (34%). Analysis of the simulations shows that N-amination favors the population of β (φ, ψ) conformations by the preceding residue due to, at least in part, a network of weak NH_2_(*i*)–CO(*i*) and NH_2_(*i*)–CO(*i*–2) hydrogen bonds. It also leads to a reduction of misfolding because of changes in the hydrogen-bonding potential. Both of these features help funnel the peptide to the folded β-hairpin structure. The conformational insights provided through this work give a firm foundation for the design of N-aminated peptide inhibitors for modulating protein–protein interactions and aggregation.

## 1 Introduction

**Figure 1:**
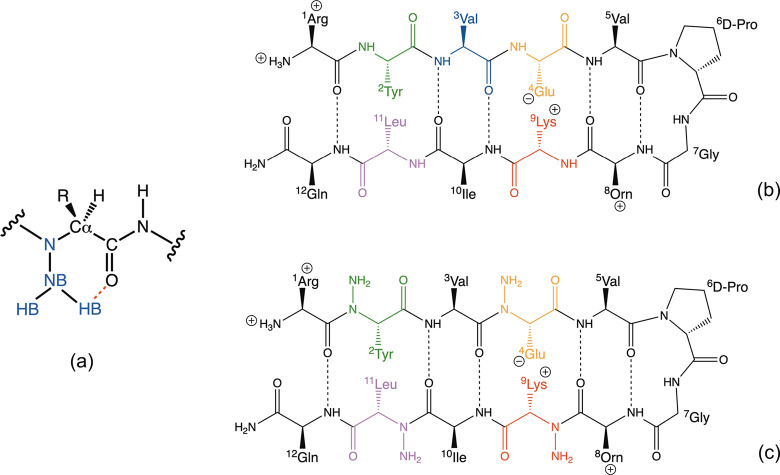
(a) Chemical structure of a residue with backbone N-amination showing the intraresidue C6 hydrogen bond NBHB(*i*)–CO(*i*) (dashed line). (b) The model peptide in β-hairpin conformation. The residues that were N-aminated in this work are shown in color. (c) Peptide with backbone N-amination on Tyr2, Glu4, Lys9, and Leu11.

An increasing range of noncanonical amino acid residues are being introduced into synthetic peptides, bringing a variety of new functionalities.^1−6^ The inclusion of backbone-substituted amino acids provides an approach for altering the main-chain conformational preferences, hydrogen-bonding capabilities, and proteolytic stability of the peptide while maintaining side-chain diversity. One such modification is the N-amination of backbone amide groups (Figure 1).^7,8^ Experimental NMR studies of peptides containing such N-aminated residues have shown that this modification can stabilize extended β-strand-like conformations.^9,10^ In addition, X-ray diffraction studies of short dipeptides and tripeptides with an N-aminated residue have identified the formation of an intraresidue hydrogen bond between the hydrazino acid NH_2_ group and the carbonyl oxygen acceptor, completing a six-membered ring, a C6 hydrogen bond (Figure 1a).^9,11^ Moreover, conformational analysis has shown that, in contrast to N-methylation, N-amination destabilizes the adoption of *cis*-peptide bonds because of electrostatic repulsions between the NH_2_ substituent and the carbonyl oxygen lone pairs.^9,10,12^

The changed hydrogen-bonding capabilities on N-amination, which preclude involvement in the normal β-sheet interactions, give the potential for designing N-aminated peptides that could block amyloid-type aggregation. This has been demonstrated using backbone N-aminated hexapeptides taken from the aggregation-prone amyloid beta sequence Aβ_16–21_.^13^ In these studies, a diaminated peptide, with N-amination at residues 3 and 5, was shown to be an effective inhibitor of the fibrilization of Aβ_42_. Furthermore, N-aminated peptides from aggregation-prone sequences from the R2 and R3 domains of tau protein have also been shown to block tau fibrilization and seeding.^14^ Another study of the possibilities of using N-aminated residues in peptide-based drug candidates has shown that N-aminated analogues of gramicidin-S have enhanced broad-spectrum microbial activity.^15^

To support the development of further applications of N-aminated peptidomimetics, one must develop a firm understanding of the conformational properties of peptides containing N-aminated residues. To accomplish this, we performed a detailed molecular dynamics (MD) simulation study of a range of N-aminated derivatives of the β-hairpin model developed by Gellman and co-workers.^16,17^ This model is a 12-residue linear peptide that contains a (d-Pro6-Gly7)-turn motif that adopts a β-turn and folds the peptide into a stable β-hairpin in water (Figure 1b). Del Valle and co-workers have used this β-hairpin peptide in their study of peptide N-amination using NMR methods to characterize four derivatives containing a single N-aminated residue and one derivative with two N-aminated residues.^9,10^

Here, MD simulations are reported for a linear and cyclic form of the original β-hairpin model peptide, the five N-aminated derivatives that have been characterized experimentally, and an additional peptide with four N-aminated residues (Figure 1b, c). Peptides Pep_N2, Pep_N9, and Pep_N11 have one N-aminated residue and Pep_N9_N11 has two N-aminated residues, all of whose NH_2_ groups point out of the β-hairpin, whereas Pep_N3 has one N-aminated residues whose NH_2_ group points into the β-hairpin. The sixth peptide, Pep_N2_N4_N9_N11, has N-amination of all four backbone amide groups that point out of the β-hairpin. For all the peptides MD simulations have been performed starting from both the folded β-hairpin structure (PepF) and an extended, unfolded peptide chain (PepE).

## 2 Methods

### 2.1 Force-Field Parameters and MD Simulation Setup

Molecular dynamics simulations were performed using the GROMOS biomolecular simulation software (http://www.gromos.net).^18,19^ The GROMOS biomolecular force field 54A7^20,21^ was used, with the force-field parameters for the amino acid ornithine and the N-aminated residues being derived manually from standard GROMOS parameters by analogy reasoning (details given in the Supporting Information).

Two 100 ns MD simulations, starting from the folded β-hairpin structure and from an extended peptide chain, were performed for each of the seven peptides: the linear β-hairpin model peptide Pep (H-Arg-Tyr-Val-Glu-Val-d-Pro-Gly-Orn-Lys-Ile-Leu-Gln-NH_2_) [Orn = ornithine], four variants of this peptide with a single residue with backbone N-amination on Tyr2 (Pep_N2), Val3 (Pep_N3), Lys9 (Pep_N9), or Leu11 (Pep_N11), one variant with two residues with backbone N-amination on Lys9 and Leu11 (Pep_N9_N11), and one variant with four residues with backbone N-amination on Tyr2, Glu4, Lys9, and Leu11 (Pep_N2_N4_N9_N11) (Figure 1b, c). In addition, a 100 ns MD simulation of a cyclic variant of the peptide PepC (cyclo[Arg-Tyr-Val-Glu-Val-d-Pro-Gly-Orn-Lys-Ile-Leu-Gln-d-Pro-Gly]) and a 50 ns MD simulation of a linear variant with residue 6 as l-Pro rather than d-Pro were also performed. For all simulations the N-terminus, Arg, Lys, and Orn side chains were protonated with charge + e.

The structure of cyclic PepC was derived from PepC experimental NOE data^9,17^ in a simulated annealing protocol for NMR structure determination^22^ in XPLOR-NIH^23,24^ (PepC NOE data are listed in Table S11 of the Supporting Information). The lowest energy structure from the calculated ensemble served as a starting structure for a 10 ns MD simulation in which NOE distance restraints were further imposed as time-averaged restraints. The final coordinates of that simulation were then used for a subsequent 100 ns long PepC production run. Similarly, the structure of Pep, the coordinates of which were generated by deleting d-Pro13 and Gly14 of PepC, first served as a starting structure for a 10 ns time-averaged distance-restrained MD simulation using the Pep^9,16^ experimental NOE data set (Table S12 of the Supporting Information). As in the case of PepC, the simulation was then continued for an additional 100 ns without any NOE restraint. Furthermore, the structure at the end of the 10 ns time-averaged distance-restrained MD simulation of Pep was also used as the starting folded β-hairpin structure for the MD simulations of all the N-aminated peptides and also the variant with l-Pro6. These simulations are referred to as the PepF simulations. In addition, an extended conformation was made from this structure by setting the backbone φ, ψ, and ω torsion angles of all the residues in the peptide to 180°. It was used as the starting structure for the MD simulations of extended Pep and of all the corresponding extended N-aminated peptides. These simulations are referred to as the PepE simulations.

In all cases the peptides were solvated in cubic boxes of SPC water molecules.^25^ Minimum image periodic boundary conditions were applied, the minimum solute-box wall distance being set to 1.2 nm. This gave 3916 and 3743 water molecules for the initial simulations of PepF and PepC respectively, between 2675 and 2730 water molecules for the simulations of the N-aminated variants starting from the folded β-hairpin structure, and between 9817 and 11242 water molecules for the simulations starting from the extended conformation. The box length ranged from 4.4 to 5.0 nm for the simulations starting from the folded peptide conformation and from 6.7 to 7.0 nm for the simulations starting from the extended conformation. For compensation for the overall positive charge of the peptides, three and two chloride ions were included in the simulations of the linear and cyclic peptides, respectively.

### 2.2 Molecular Dynamics Simulations

For each simulation an initial equilibration scheme consisting of five 20 ps simulations at 60, 120, 180, 240, and 300 K was used at constant volume. During the first 80 ps of this equilibration the solute atoms were harmonically restrained to their positions in the initial structure with force constants of 25000, 2500, 250, and 25 kJ mol^–1^ nm^–2^, respectively. After equilibration, the simulations were run at 300 K and 1 atm pressure using the weak-coupling algorithm,^26^ with relaxation times of τ_T_ = 0.1 ps and τ_p_ = 0.5 ps and an isothermal compressibility of 4.575 × 10^–4^ (kJ mol^–1^ nm^–3^)^−1^. The solute and solvent were separately coupled to the heat bath. The SHAKE algorithm^27^ was used to constrain bond lengths with a relative geometric tolerance of 10^–4^, allowing for an integration time step of 2 fs. The center of mass motion of the system was removed every 1000 time steps. Nonbonded interactions were calculated using a triple-range cutoff scheme with cutoff radii of 0.8 and 1.4 nm. Interactions within 0.8 nm were evaluated every time step, and intermediate interactions were updated every fifth time step. To account for the influence of the dielectric medium outside the 1.4 nm cutoff sphere, a reaction-field force^28^ with a dielectric permittivity of 61^29^ was used. In the initial 10 ns simulations with the NOE restraint,^30^ the force constant for the NOE distance restraint was 10^3^ kJ mol^–1^ nm^–2^, the memory relaxation time was 5 ps, and *r*^–3^ averaging was used.

### 2.3 Analysis of the MD Simulations

Analysis was based on solute configurations saved every 5 ps. For the root-mean-square deviation (RMSD) calculations, clustering and hydrogen bond, NOE, and time series analysis, the GROMOS++ software was used.^31^ Atom-positional RMSD values were calculated for the backbone N, CA, and C atoms of residues 2–11 after superposition of the molecular centers of mass and a rotational least-squares fit of the positions of these atoms. Conformational clustering was performed using the algorithm of Daura et al.^32^ and the backbone N, CA, and C atoms of residues 2–11. The RMSD cutoff used to determine the structures belonging to a single cluster was 0.1 nm. The overall twist of the β-hairpin was analyzed through the simulations by calculating the time series of a torsion angle defined by the positions of the atoms 1C–12N–8C–5N. In the comparison with the experimental NOE distance restraints, the pseudoatom corrections of Wüthrich et al.^33^ were used, and the interhydrogen distances were calculated as ⟨*r*^–6^⟩^–1/6^ because for motions such as peptide plane flips the overall molecular tumbling is fast compared to the internal motions.^34,35^ Hydrogen bonds were identified according to geometric criteria. Two definitions were used for hydrogen bonds: a medium-strong hydrogen bond was assumed to exist if the hydrogen-acceptor distance was smaller than 0.25 nm and the donor-hydrogen-acceptor angle was larger than 135°, whereas a weak hydrogen bond was assumed to exist if the hydrogen-acceptor distance was smaller than 0.32 nm and the donor-hydrogen-acceptor angle was larger than 90°.^36^

## 3 Results and Discussion

### 3.1 Simulations from the Folded Conformation

**Figure 2:**
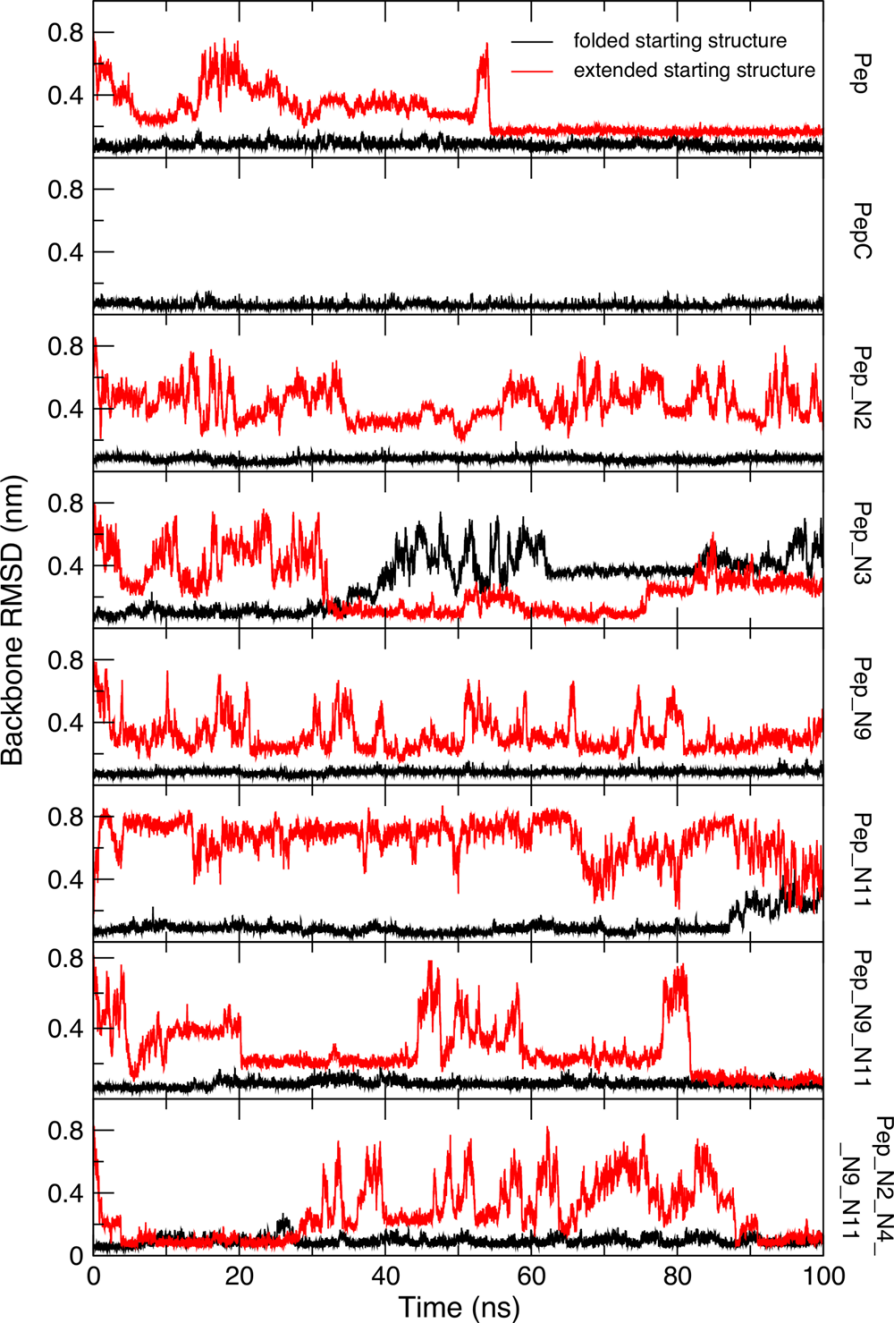
Backbone (N, CA, and C) atom-positional root-mean-square deviation for residues 2–11 of the simulated conformers compared to the folded β-hairpin structure as a function of simulation time. For each peptide the data for the simulations starting from the folded β-hairpin and from the extended structure are shown in black and red, respectively.

**Figure 3:**
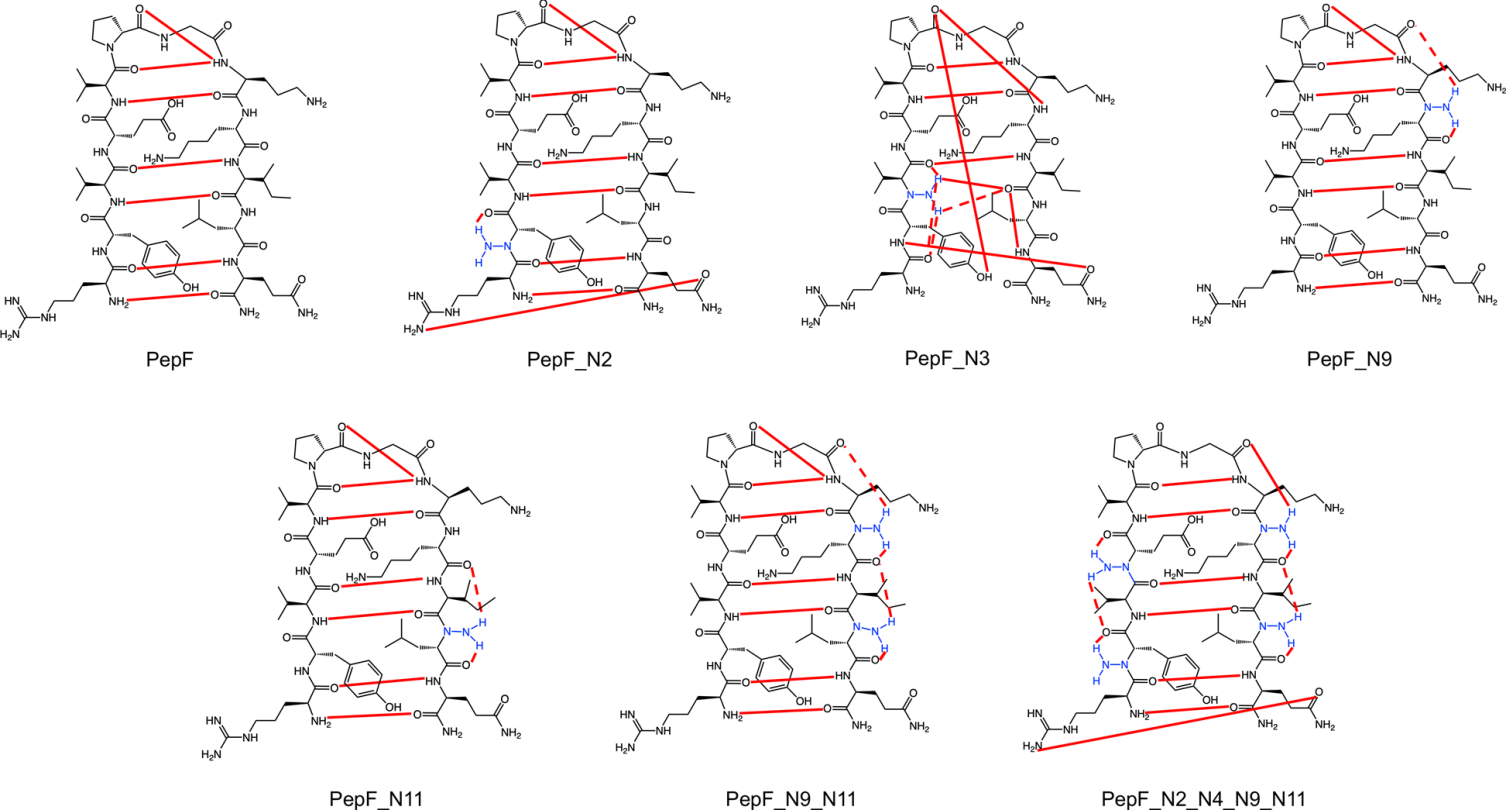
Main hydrogen bonds (population >10%) present in the 100 ns simulations of the β-hairpin peptides starting from a folded β-hairpin structure. Solid red lines show medium-strong (H-acceptor distance <0.25 nm and donor-H-acceptor angle >135°) hydrogen bonds and dashed red lines show weak (H-acceptor distance <0.32 nm and donor-H-acceptor angle >90°) hydrogen bonds. In the simulations the N-terminus, Arg, Lys, and Orn side chains are protonated.

One hundred nanosecond MD simulations were run for the model β-hairpin peptide in both linear and cyclic form, starting from a folded β-hairpin structure calculated from the experimental NOE data (simulations PepF and PepC). Both peptides show low atom positional RMSD values compared to the folded starting structure for the backbone of residues 2–11 throughout the simulation (mean RMSD 0.082 and 0.060 nm for PepF and PepC respectively; Figure 2). The main hydrogen bonds that define the β-hairpin (3NH–10O, 5NH–8O, 10NH–3O, and 12NH–1O) have populations greater than 70% in both simulations (Table 1 and Figure 3). The hydrogen bond populations are similar for both peptides except that the cyclic peptide has higher populations of hydrogen bonds between residues 1 and 12, which are at the termini of the linear peptide. Because PepC is considered to be fully folded (NMR chemical shift differences between linear and cyclic peptides have been used to estimate the fraction of folded conformers in experimental studies^9,16,17^), the close similarity of the conformational characteristics of PepF and PepC in the unrestrained simulations is an important observation.

**Table 1:** Hydrogen-Bond Populations (in %) in the 100 ns Simulations of the β-Hairpin Peptides Starting from a Folded β-Hairpin Structure^a^

	peptide							
hydrogen bond	PepF	PepC	PepF_N2	PepF_N3	PepF_N9	PepF_N11	PepF_ N9_N11	PepF_N2_ N4_N9_N11
1NH1–12O	12	57	16	3	11	12	13	21
1NH2–12O	12		17	3	11	13	13	18
1NH3–12O	12		17	3	11	12	13	20
1HE–12OE1	6	10	6	1	6	3	4	4
1HH21–12OE1	8	3	14	2	7	7	6	10
2NH–12OE1				16				
2OH–6O				17				
3NH–10O	95	90	94	15^b^	97	83	93	95
5NH–8O	74	77	77	25	81	67	82	74
8NH–5O	36	33	36	13	47	36	49	57
8NH–6O	16	14	18	5	13	16	10	6
9NH–6O				10				
9NH–7O	2			3	5^b^	2	7^b^	15^b^
10NH–3O	81	86	87	24	82	75	85	86
12NH–1O	76	92	77	6	73	60	60	67
12NH–10O	1	1		15	1	2	1	1

a The hydrogen bonds were identified using the definition for medium-strong hydrogen bonds: H-acceptor distance <0.25 nm and donor-H-acceptor angle >135°. Only hydrogen bonds with a population >10% in at least one simulation are included. Populations <1% are excluded.

b The hydrogen bond donor residue is N-aminated, and so the hydrogen bond donor is the NH_2_ group rather than the amide NH. The value listed is the highest of the hydrogen bond populations of NH1 and NH2 in the NH_2_ group.

The PepF simulation agrees well with the available experimental NOE data (Table S12 in the Supporting Information). For the 21 experimental NOE restraints, there is only one violation (Gln12HE21–Ile10HG2, NOE violation 0.067 nm). This small violation probably reflects the fact that the dynamics of the side chains of Ile10 and Gln12 may not be fully sampled in the 100 ns simulation. An MD simulation starting from the same β-hairpin fold but with d-Pro7 replaced with l-Pro7 unfolded within 30 ns, reflecting the importance of the d-Pro residue in the β-turn conformation. These results agree with experimental data that show that the l-Pro derivative is fully unfolded.^9,16^

One hundred nanosecond MD simulations were then run for the six derivatives containing one or more N-aminated residues starting from the folded β-hairpin structure. The effects of N-amination vary significantly depending on the position of the modified amino acid. The peptides in the PepF_N2, PepF_N9, PepF_N9_N11, and PepF_N2_N4_N9_N11 simulations all remain folded with backbone atom positional RMSD values similar to those seen in the PepF simulation (Figure 2). N-Amination of Leu11 near the C-terminus leads to significant fluctuations in the ψ angle of Leu11, giving fraying to the ends of the β-hairpin and reducing the population of the 12NH–1O hydrogen bond in the peptide, particularly in the PepF_N11 and PepF_N9_N11 simulations. Some unfolding of the peptide is also seen at the end of the PepF_N11 simulation with the loss of the 3NH–10O and 10NH–3O hydrogen bonds. However, N-amination of Tyr2 near the N-terminus results in a slight increase in the population of hydrogen bonds between residues 1 and 12 compared to the PepF simulation. In contrast to other derivatives, N-amination of Val3, a residue whose NH is involved in one of the key β-hairpin hydrogen bonds, gives unfolding of the β-hairpin after about 40 ns in the PepF_N3 simulation. These results are in agreement with the experimental NMR chemical shift and NOE data, which show that all the N-aminated derivatives of the peptide, except for the peptide with N-amination of Val3, adopt a stable β-hairpin fold with the same hydrogen-bonding register. When the NOE distances calculated from the PepF_N2, PepF_N9, PepF_N11, and PepF_N9_N11 simulations are compared to the experimentally derived average NOE atom–atom distance upper bounds, only one violation greater than 0.05 nm has been observed (violation of 0.083 nm for 2HD–10HA in Pep_N2), indicating excellent agreement with the experimental data also for the N-aminated β-hairpin peptides. Morover, Sarnowski et al. have shown that the diastereotopic separation of the Gly7Hα chemical shifts is much reduced in the case of the peptide with N-amination of Val3, indicating a decreased folded population that has also been observed in our simulations.^9^ A detailed comparison of the simulations and the experimental NOE data is given in Tables S13–S16 of the Supporting Information.

In all cases weak intraresidue C6 hydrogen bonds between the NBHB2 group and the backbone carbonyl oxygen are seen for the N-aminated residues, although the populations of these hydrogen bonds vary significantly between the different N-amination sites (Table 2). The highest populations (96–97%) are seen for N-aminated Lys9, whereas N-aminated Val3 and Glu4 show the lowest populations of 22% and 27%, respectively. In the simulations, weak hydrogen bonds are also seen between the NBHB1 group of the N-aminated residue “*i*” and the carbonyl oxygen of residue “*i*–2”. PepF_N2_N4_N9_N11 shows the highest population of these hydrogen bonds (43–54%). The intraresidue C6 hydrogen bonds have been observed experimentally in crystal structures of a short dipeptide^11^ and a tripeptide^9^ containing N-aminated residues, although these peptides are not long enough to show the NH_2_(*i*)–CO(*i*–2) hydrogen bonds also seen in the simulations. Note that the criteria used for identifying the hydrogen bonds from the N-amination groups are those defined by Gilli and Gilli for identifying weak hydrogen bonds (H-acceptor distance <0.32 nm and donor-H-acceptor angle >90°).^36^ The C6 hydrogen bonds in the crystal structure of the N-aminated tripeptide show donor-H-acceptor angles in the range 112–130°.^9^ These angles are lower in value than those used in the standard hydrogen bond definition (H-acceptor distance <0.25 nm and donor-H-acceptor angle >135°), confirming the role of weak hydrogen bonds in these N-aminated systems.

**Table 2:** Populations of Weak Hydrogen Bonds (in %) Involving the N-Amination Group in the 100 ns Simulations of the β-Hairpin Peptides Starting from Both the Folded β-Hairpin Structure and the Extended Conformation^a^

	peptides and N-amination sites											
	N2		N3		N9		N11		N9_N11		N2_N4_ N9_N11	
hydrogen bond	PepF	PepE	PepF	PepE	PepF	PepE	PepF	PepE	PepF	PepE	PepF	PepE
2NBHB2–2O	69	26									57	26
3NBHB1–1O			40	34								
3NBHB1–10O			15	27								
3NBHB2–3O			22	53								
3NBHB2–1O			11	4								
3NBHB2–8O				10								
3NBHB2–10O			25	23								
4NBHB1–2O											54	54
4NBHB2–4O											27	20
9NBHB1–7O					28	28			31	38	43	38
9NBHB2–9O					96	44			97	77	97	58
9NBHB2–12OE1										12		
11NBHB1–9O							34	51	32	46	51	44
11NBHB2–11O							35	15	34	3	36	21

a The hydrogen bonds were identified using the definition for weak hydrogen bonds: H-acceptor distance <0.32 nm and donor-H-acceptor angle >90°. Only hydrogen bonds with a population >10% in at least one simulation are included. Populations <1% are excluded.

**Figure 4:**
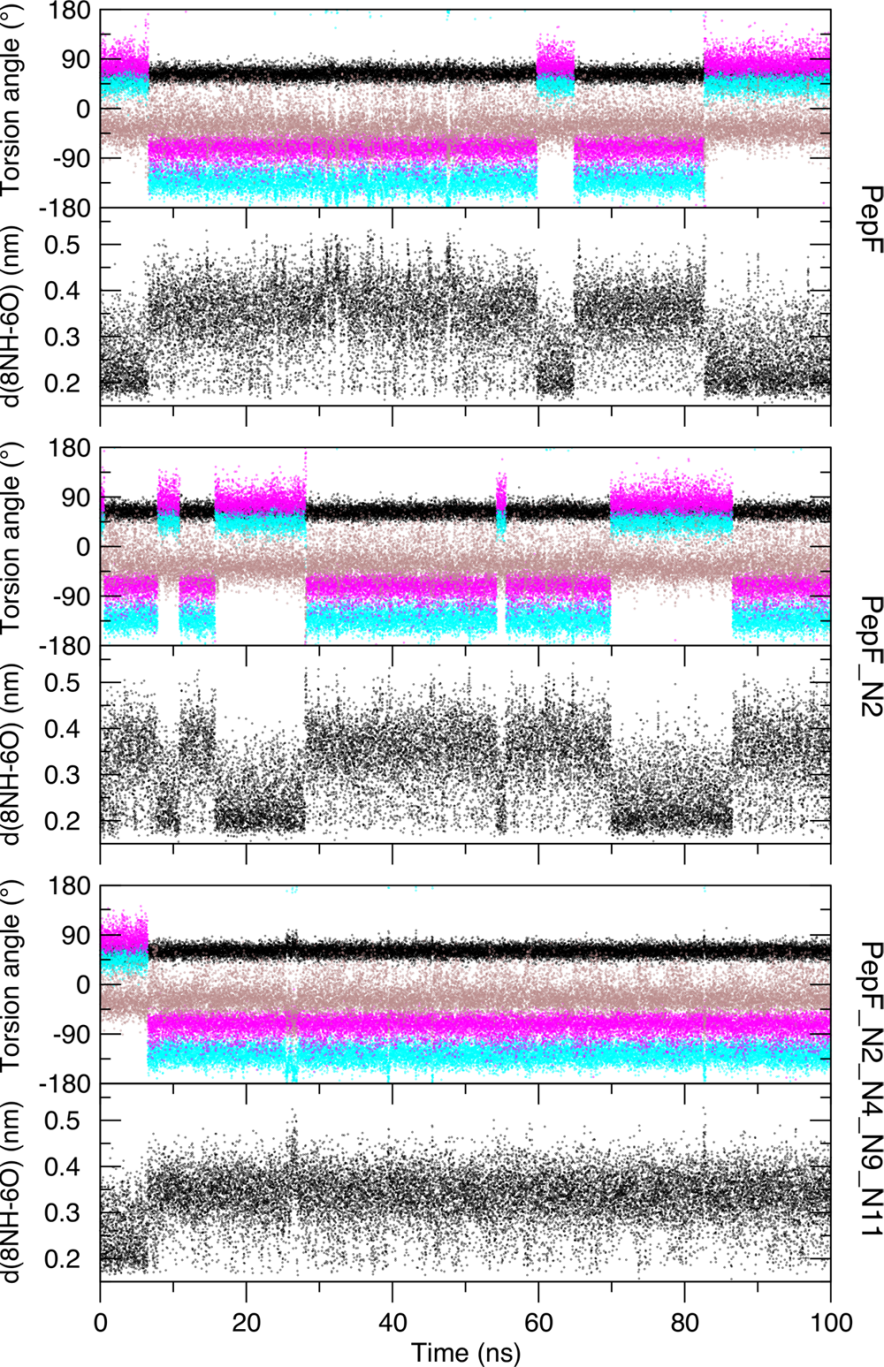
Variations in the backbone torsion angles of residues d-Pro6 (φ, black; ψ, cyan) and Gly7 (φ, magenta; ψ, brown) and the 8NH–6CO distance (black) in the peptide as a function of time in the simulations PepF (top two panels), PepF_N2 (middle two panels), and PepF_N2_N4_N9_N11 (bottom two panels).

In this study we have also observed a significant effect of N-amination on the torsional space of the β-hairpin backbone, especially on its β-turn. Figure 4 shows that residues d-Pro6–Gly7 adopt a mixture of type I′ and type II′ β-turns. Regular type I′ β-turns have (φ, ψ) torsion angles of approximately (60°, 30°) and (90°, 0°) for residues *i*+1 (d-Pro6) and *i*+2 (Gly7), respectively, whereas type II′ β-turns have (φ, ψ) torsion angles of approximately (60°, −120°) and (−80°, 0°) for residues *i*+1 and *i*+2, respectively.^37,38^ X-ray structures of peptides containing the d-Pro-Gly sequence predominantly show a type II′ β-turn, although some examples with a type I′ β-turn have been reported.^1,39,40^ Interconversion between type I′ and type II′ β-turns occurs via a peptide plane flip,^41,42^ and in the simulations of PepF and PepF_N2 the d-Pro 6 – Gly 7 sequence makes four and nine of these peptide plane flip transitions, respectively, during the 100 ns simulations. However, only one transition is seen for PepF_N2_N4_N9_N11 (Figure 4, bottom). An increased population of the 8NH–6O hydrogen bond is seen when the type I′ turn is populated as this distance is shorter in that conformation (Figure 4), whereas the type II′ β-turn appears to slightly favor the 9NBHB1–7O hydrogen bond. Experimentally, NMR data have shown that peptide plane flip transitions between type I′ and type II′ β-turns occur in some peptides containing a d-Pro-d-Ala motif, where there is a preference for a type I′ β-turn conformation.^43^ However, in these cases the rate of interconversion is on the millisecond time scale, slow exchange being observed in the NMR spectra.^44^ Interconversion is therefore significantly slower than that seen here for the d-Pro-Gly sequence.

The β-hairpin starting structure for the MD simulations contained a type I′ β-turn (both type I′ and type II′ β-turns are consistent with the NOE data), but all the peptides show a preference for the type II′ β-turn in the simulations, in agreement with the recognized preference of the d-Pro-Gly sequence.^1,39,40^ Particularly, N-amination of residues toward the center of the peptide gives an increased population of the type II′ β-turn. For example, the populations of the type II′ β-turn for the peptides in the PepF, Pep_N9, Pep_N9_11, and Pep_N2_N4_N9_N11 simulations are 71%, 80%, 84%, and 94%, respectively (values calculated from the ψ torsion angle populations of d-Pro6). The cyclic peptide PepC shows a higher type II′ β-turn population (82%) than the linear PepF (71%) for residues Gly6 and d-Pro7. d-Pro13 and Gly14 in peptide PepC adopt only a type II′ β-turn in the simulation.

Changes in the (φ, ψ) values of the N-aminated residues also affect the twist of the β-hairpin. The β-hairpin in PepF has a right-handed twist, with a similar twist angle to that seen in the cyclic PepC (mean twist angles in the simulations defined by the 1C-12N-8C-5N torsion angle are 87.2° and 88.3° for PepF and PepC, respectively). This right-hand twist in antiparallel β-sheets is well recognized and has been attributed to intrastrand electrostatic and van der Waals interactions and to interstrand hydrogen bonding.^45−48^ N-Amination, particularly of residues toward the center of the peptide, leads to a flattening of the β-hairpin. So, for example, the mean twist angles for the peptide in the PepF_N9, PepF_N9_N11, and PepF_N2_N4_N9_N11 simulations are 84.0°, 69.4°, and 56.5°, respectively. This flattening comes from small changes in the (φ, ψ) values of the N-aminated residues and appears to result in the reduced preference to adopt the type I′ β-turn on N-amination. For example, in the PepF_N2_N4_N9_N11 simulation the starting structure adopts a type I′ turn, but the peptide swaps to a type II′ β-turn after 6.5 ns and continues in the type II′ turn for the rest of the simulation (Figure 4). Prior to 6.5 ns the mean twist angle in the β-hairpin is 79.7°, and then after that point the mean twist angle is 54.9°.

### 3.2 Simulations from an Extended Conformation

One hundred nanosecond MD simulations were also run for all the peptides, except PepC, starting from an unfolded extended structure. The backbone atom positional RMSD values compared to the folded β-hairpin structure for residues 2–11 for all the peptides in these simulations are shown in Figure 2. In interpreting these data, and the following analysis of torsion angle and hydrogen bond populations, we note that none of the simulations reported here are long enough to draw definite conclusions about the relative stability of the folded and unfolded states or to assess the statistical uncertainty of hydrogen bond populations. For this, much longer simulation times with multiple folding and unfolding events would be required. Interestingly enough, even within the 100 ns simulations reported here, three of the peptides fold into a β-hairpin. Peptide folding in the PepE_N2_N4_N9_N11 simulation is most extensive with the key hydrogen bonds 3NH–10O, 5NH–8O, 10NH–3O, and 12NH–1O having populations of 31%, 27%, 34%, and 17%, respectively (Table 3). As can be seen from Figure 2, this peptide also makes a number of folding and unfolding transitions. It folds first in the simulation from 3.8 to 27.5 ns. It then unfolds and folds again briefly from 88 to 88.5 ns and then refolds from 91 ns until the end of the simulation (mean backbone RMSD during folded periods 0.095 nm).

**Table 3:** Hydrogen Bonds Populations (in %) in the 100 ns Simulations of the β-Hairpin Peptides Starting from the Extended Conformation^a^

	peptide						
hydrogen bond	PepE	PepE_N2	PepE_N3	PepE_N9	PepE_N11	PepE_ N9_N11	PepE_N2_N4_N9_N11
1NH1–12O			4	1		1	6
1NH2–12O			4	1		1	5
1NH3–12O			4	1		1	6
3NH–1O	5		6^b^	82	40	2	2
3NH–8O			6^b^			30	
3NH–10O			14^b^			14	34
3NH–11O	23						
5NH–2O		2		51	27		
5NH–8O			25		2	17	27
5NH–9O	50						
7NH–10O		6			27		
8NH–5O			17	10	4	13	21
8NH–6O	7	3	5	20	6	4	6
9NH–7O	39	3	5	10^b^	6	13^b^	13^b^
10NH–3O			22			21	31
10NH–8O	8	9	14	4	27	3	3
11NH–3O	51						
12NH–1O			10				17

a The hydrogen bonds were identified using the definition for medium-strong hydrogen bonds: H-acceptor distance <0.25 nm and donor-H-acceptor angle >135°. Only hydrogen bonds that define the β-hairpin fold or those with a population >20% in at least one simulation are included. Populations <1% are excluded.

b The hydrogen bond donor residue is N-aminated, and so the hydrogen bond donor is the NH_2_ group rather than the amide NH. The value listed is the highest of the hydrogen bond populations of NH1 and NH2 in the NH_2_ group.

The peptide in the PepE_N3 simulation folds from 33 to 50.5 ns; it partially unfolds but retains the 8NH–5O hydrogen bond to some extent and then folds again from 58.5 to 75.7 ns (mean backbone RMSD during folded periods is 0.1 nm). In this simulation the hydrogen bond 3NBHB1–10O from the N-aminated group has a population of 14%, and the other key β-hairpin hydrogen bonds 5NH–8O, 10NH–3O, and 12NH–1O have populations of 25%, 22%, and 10%, respectively. It is interesting that N-amination of Val3 appears to promote peptide folding in the PepE_N3 simulation because this peptide does not stay folded in the PepF_N3 simulation starting from the folded β-hairpin, and experimental studies show this peptide has a decreased folded population. The NH of Val3 forms one of the key β-hairpin hydrogen bonds and maybe the protrusion of the NH_2_ group from the peptide backbone, or the presence of two NH groups rather than the single amide NH, increases the likelihood of the 3NBHB1–10O hydrogen bond formation. Note that the population of the 3NBHB2–3O hydrogen bond is higher in the PepE_N3 simulation than in the PepF_N3 simulation, but its population is low in the parts of the PepE_N3 simulation where the peptide is folded.

The peptide in the PepE_N9_N11 simulation folds briefly between 5.5 and 6 ns. It folds again at 84.1 ns and remains folded until the end of the simulation (mean backbone RMSD during folded periods, 0.105 nm). In this simulation the population of the β-hairpin hydrogen bonds 3NH–10O, 5NH–8O, and 10NH–3O are 14%, 17%, and 21%, respectively, but the 12NH–1O hydrogen bond is not observed.

**Figure 5:**
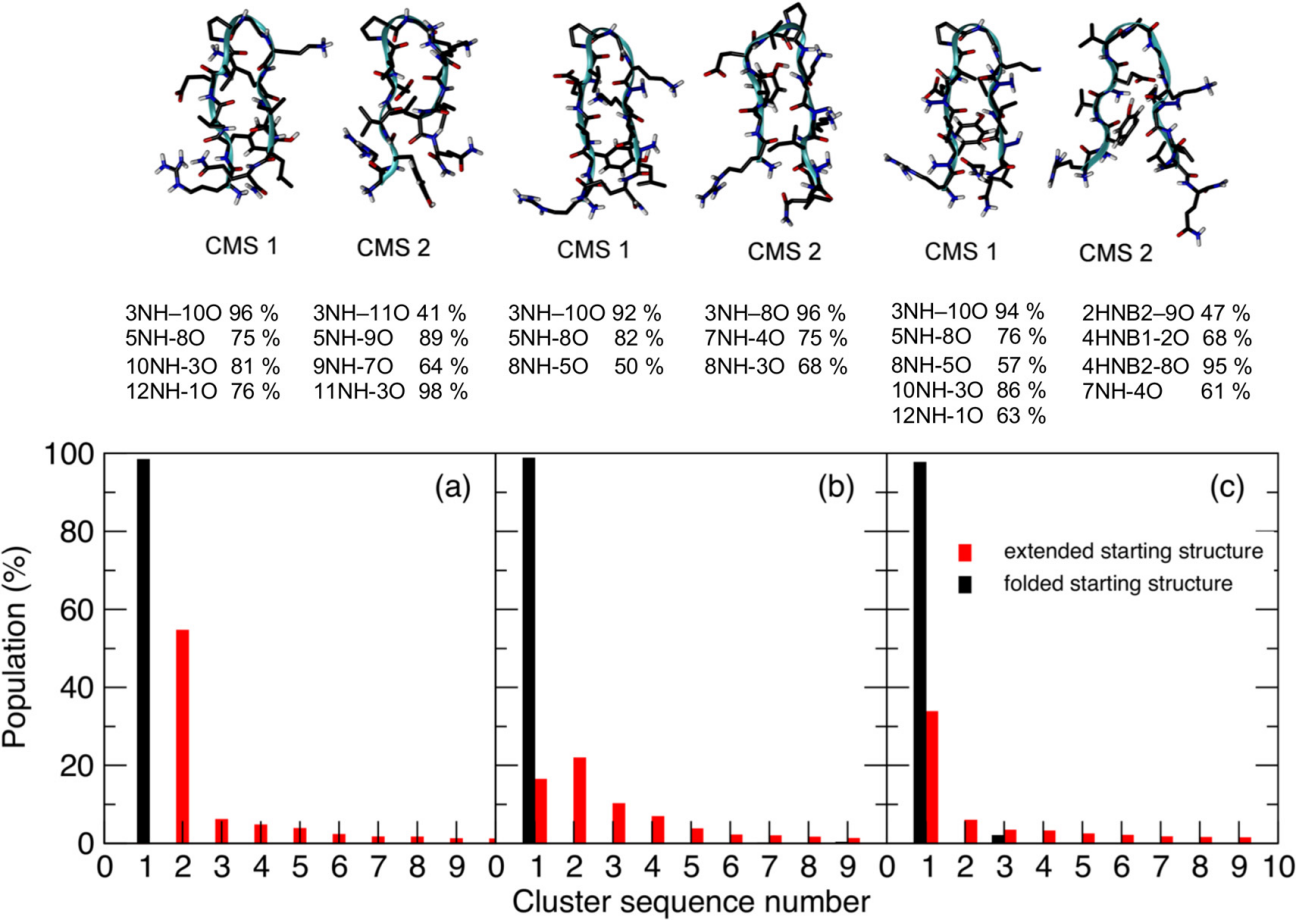
Conformational analysis of the combined trajectories of the peptides PepF and PepE (a), PepF_N9_N11 and PepE_N9_N11 (b), and PepF_N2_N4_N9_N11 and PepE_N2_N4_N9_N11 (c). Upper part: The central structures of the two most populated clusters for each of the combined trajectories along with the occurrence of the major hydrogen bonds. Lower part: Contribution of the individual trajectories to the clusters.

As hydrogen-bonding analysis and backbone root-mean-square deviations indicate that the conformational spaces sampled by PepF_N2_N4_N9_N11 and PepE_N2_N4_N9_N11 probably overlap, a combined conformational clustering analysis of the two trajectories was performed to investigate this issue. With a cutoff RMSD of 0.1 nm for the backbone of residues 2–11, the largest cluster indeed contains the folded structures, consisting of 98% and 34% of the conformers from the PepF_N2_N4_N9_N11 and PepE_N2_N4_N9_N11 simulations, respectively. (Table S25 in the Supporting Information gives details of the hydrogen bonds in the peptide in the different clusters identified.) The identification of the overlap between the conformers in the two simulations confirms that the peptide PepE_N2_N4_N9_N11 folds into the correct β-hairpin structure (Figure 5). The backbone (φ, ψ) torsion angles are closely similar in the conformers of the folded cluster from the two simulations with the mean twist angles of the β-hairpin in the peptide being 58.1° and 56.0° for the PepF_N2_N4_N9_N11 and PepE_N2_N4_N9_N11 simulations, respectively. Moreover, during the folded periods of PepE_N2_N4_N9_N11 simulation, the peptide adopts only the type II′ and not the type I′ β-turn.

Combined clustering analysis has also been performed for the PepF and PepE trajectories as well as for the PepF_N9_N11 and PepE_N9_N11 trajectories. Although the conformational spaces visited by PepF_N9_N11 and PepE_N9_N11 do show some overlap, PepF and PepE populate structures with different relative registers for the two β-strands. The misfolded form of PepE populates the second largest conformational cluster of the combined ensemble. In this cluster the peptide is characterized by the hydrogen bonds 11NH–3O (98%), 5NH–9O (89%), 9NH–7O (64%), and 3NH–11O (41%) and adopts a γ-turn (further details given in Table S19 in the Supporting Information). For PepE_N9_N11 simulation the misfolded form is found in cluster 2 (22%) and is populated from approximately 20 to 44.5 ns. The conformers in this cluster contain the hydrogen bonds 3NH–8O (96%), 7NH–4O (75%), 8NH–3O (68%), 10NH–1O (60%), and 1NH–10O (21%). Here, the β-turn is shifted by one residue compared to the experimental fold, so the 7NH–4O hydrogen bond is observed rather than the 8NH–5O hydrogen bond (Table S22). Note that we refer to the conformers with different hydrogen-bonding registers in the PepE and PepE_N9_N11 simulations as misfolded forms because the experimental NOE data available for both of these peptides include the NOE 2Hα–11Hα. These NOE data confirm that the same β-hairpin fold, with the same hydrogen-bonding register as is seen in PepC, is observed experimentally for these peptides.

**Figure 6:**
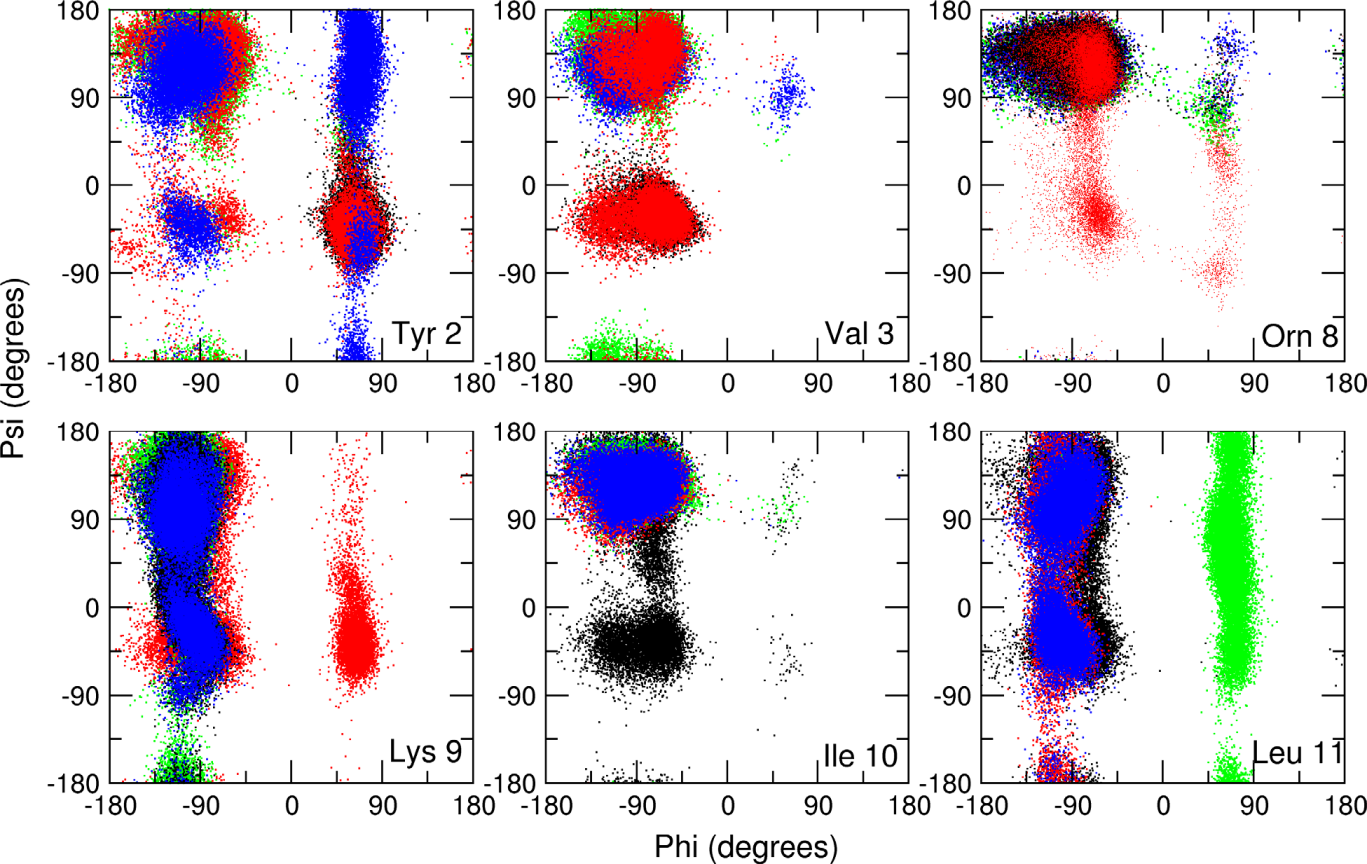
Ramachandran plots showing the populations of main chain (φ, ψ) torsion angles for residues Tyr2, Val3, Orn8, Lys9, Ile10, and Leu11 of the peptide in the PepE_N9 (black), PepE_N11 (red), PepE_N9_N11 (green), and PepE_N2, N4_N9_N11 (blue) MD simulations.

For an understanding of the reasons for the increased folding seen when a number of residues with amide groups pointing out of the β-hairpin fold are N-aminated, particularly in the PepE_N2_N4_N9_N11 simulation, the backbone torsion angles in the peptide were followed through the simulation (Figure 6). N-Amination of a given residue *i* was found to lead to the favoring of β-conformations by the preceding residue *i*–1. For example, in the PepE_N9 simulation Orn8 has its (φ, ψ) torsion angles confined to the β-region, but the residues Lys9 (N-aminated), Ile10, and Leu11 have disordered torsion angles and Val3 adopts conformations in the α-region of the (φ, ψ) space. In contrast, in the PepE_N11 simulation residues Val3, Orn8, Lys9, and Leu11 (N-aminated) have disordered (φ, ψ) torsion angles, but Ile10 has (φ, ψ) torsion angles confined to the β-region. Furthermore, in the PepE_N9_N11 and PepE_N2_N4_N9_N11 simulations, Val3, Orn8, and Ile10 have almost all their (φ, ψ) torsion angles confined to the β-region, whereas the N-aminated residues Tyr2, Glu4, Lys9, and Leu11 mostly populate a mixture of α, β, and other (φ, ψ) conformations.

This ordering of the (φ, ψ) conformation in the peptide for a residue preceding an N-amination site in the simulations from the extended conformation reflects, at least in part, the population of weak NBHB2(*i*)–O(*i*–2) hydrogen bonds together with the intraresidue C6 hydrogen bonds (Table 2). For example, in the PepE_N2_N4_N9_N11 simulation the weak hydrogen bonds 4NBHB1–2O, 9NHHB1–7O, and 11NBHB1–9O in the peptide have populations of 54%, 35%, and 44%, respectively (Table 2). The strong preference for (φ, ψ) conformations in the β-region for residues preceding the N-amination sites, and the consequent reduction in the conformational space explored, will be responsible to some extent for the folding of the peptide into the β-hairpin seen in the PepE_N9_N11 and PepE_N2_N4_N9_N11 simulations.

Another important feature is likely to be the destabilization of misfolded forms due to the changed hydrogen bond capabilities on N-amination. In the PepE simulation, where the peptide folds into a γ-turn, two of the key hydrogen bonds (11NH–3O, 9NH–7O) involve amide groups that are N-aminated in the peptides in the PepE_N9_N11 and PepE_N2_N4_N9_N11 simulations, while a third hydrogen bond involves the carbonyl oxygen of N-aminated Lys9 (5NH–9O), which will be involved in the intraresidue C6 hydrogen bond. This misfolded form is therefore likely to be destabilized in these derivatives. Similarly, the misfolded form observed in the PepE_N9_N11 simulation is likely to be less favored in the Pep_N2_N4_N9_N11 peptide, as the intraresidue C6 hydrogen bond of N-aminated Lys4 will compete with the formation of the 7NH–4O hydrogen bond. Of all the N-aminated derivatives studied experimentally, the diaminated peptide Pep_N9_N11, which shows some folding in the simulation, has the highest mean folded fraction from an analysis of Hα chemical shift data.^9^ The four-substituted derivative could not be studied experimentally because of synthetic challenges,^9^ but the data reported here suggest that it would show the greatest stabilization of the β-hairpin fold compared to that of the parent peptide of all the derivatives.

## 4 Conclusions

N-Amination changes the backbone conformational preferences and hydrogen-bonding potential of the modified residue. The MD simulations reported here have first characterized the consequences of these effects on a folded β-hairpin peptide structure. The effects are very sequence-dependent. N-Amination of Val3, a residue involved in interstrand hydrogen bonds in the β-hairpin, resulted in the unfolding of the β-hairpin after about 40 ns in the simulation. However, the introduction of N-aminated residues at positions that were pointing out of the β-hairpin structure did not disrupt the hairpin fold, although some fraying at the termini were seen on the N-amination of Leu11 and some unfolding was observed at the end of the PepF_N11 simulation. The introduced N-aminated groups formed weak intraresidue NH_2_(*i*)–CO(*i*) C6 hydrogen bonds and also NH_2_(*i*)–CO(*i*–2) hydrogen bonds. Some subtle changes were seen in the β-hairpin structure as the number of N-amination sites increased. In particular, the right-handed twist of the β-hairpin was reduced, and the adoption of a type II′ compared to that of a type I′ β-turn was increasingly favored.

The second set of MD simulations presented here probed the effects of N-amination on the folding of the model peptide from a fully extended conformation. These simulations showed that the strategy of N-aminating alternate residues along a peptide chain can favor a β-strand conformation. This is promoted by the conformation of the preceding residue being restricted to the β-region of the (φ, ψ) space, presumably because of the network of weak intraresidue C6 hydrogen bonds and also NH_2_(*i*)–CO(*i*–2) hydrogen bonds. In addition, the simulations show that the strategy can disfavor alternate misfolded hydrogen-bonded forms. In the case of the peptide studied here, N-amination of four residues resulted in 34% of the conformers in a 100 ns simulation, adopting the fully folded β-hairpin, whereas unsubstituted or singly substituted peptides, in general, misfolded or did not fold into the β-hairpin structure at all during the 100 ns simulations.

This work has illustrated the strength of complementing experimental investigations with MD simulation studies. The MD simulations have been able to clearly identify factors that give rise to the experimentally observed increased stability of the β-hairpin fold on N-amination. Moreover, the MD simulations allowed us to study a derivative with more N-amination groups than had been studied experimentally because of synthetic challenges.^9^ The results for this four-substituted derivative are particularly important, showing the strength of the approach of N-aminating alternate residues along a peptide sequence in stabilizing a β-strand conformation. This strategy results in a stable folded β-hairpin where all the backbone amides pointing out of the β-strands are N-aminated. Because the residues will be unable to participate in normal interstrand β-sheet hydrogen bonds, the possibilities for higher order β-sheet-like aggregation are disrupted. Promising results have already been reported from using peptides containing N-aminated residues to inhibit the fibrilization of Aβ_42_ and tau protein.^13,14^ N-Aminated residues in peptides also have the potential to act as inhibitors of β-strand mediated protein–protein interactions, and the MD simulations reported here reveal an important understanding that contributes to the design of these systems.

## Data and Software Availability

All the structures and MD trajectories mentioned in the article are available from the corresponding authors upon reasonable request. The following freely available software was used in this work: GROMOS biomolecular simulation software http://www.gromos.net; XPLOR-NIH https://nmr.cit.nih.gov/xplor-nih; VMD for molecular visualization https://www.ks.uiuc.edu/Research/vmd/; xmgrace https://plasma-gate.weizmann.ac.il/pub/grace/.

## Supporting Information Available

Figures of building blocks with force field parameters for ornithine and the N-aminated leucine, comparison of the MD simulation trajectories with experimental NOE data, and hydrogen bond analysis of the clusters from the trajectories PepF, PepE, PepF_N9_N11, PepE_N9_N11, PepF_N2_N4_N9_N11, and PepE_N2_N4_N9_N11 (PDF)
GROMOS molecular topology building blocks for ornithine and for the N-aminated Tyr, Val, Glu, Lys, and Leu (ZIP)

## Abbreviations

MD: molecular dynamics
NMR: nuclear magnetic resonance
NOE: nuclear Overhauser effect
RMSD: root-mean-square deviation
SPC: simple point charge
