# Supplementary material for: Backbone N-Amination Promotes the Folding of β-Hairpin Peptides via a Network of Hydrogen Bonds
Source: J Chem Inf Model. 2022 Jul 11;62(24):6704–14. doi: 10.1021/acs.jcim.2c00516 (PMC9795546; doi:10.1021/acs.jcim.2c00516)
Supplement: Supplementary file 1 — ci2c00516_si_001.pdf [file ci2c00516_si_001.pdf]

Supporting Information

Backbone N-amination promotes the folding of β-hairpin peptides via a network of hydrogen bonds

Jožica Dolenc[a]*, Esme J. Haywood*[b]*, Tingting Zhu*[b] *and Lorna J. Smith*[b]*

[a] Chemistry | Biology | Pharmacy Information Center, ETH Zurich, Zurich, Switzerland

[b] Department of Chemistry, University of Oxford, Inorganic Chemistry Laboratory, South Parks Road, Oxford OX1 3QR, United Kingdom

1. Molecular topology building blocks

1.1. ORNH Building Block

Figure S1. ORNH building block with atom numbering, GROMOS integer atom codes (IAC) and partial charges: $^{\text{atom number}}X^{IAC}_{\text{partial charge}}$. Color boundaries indicate different charge groups.

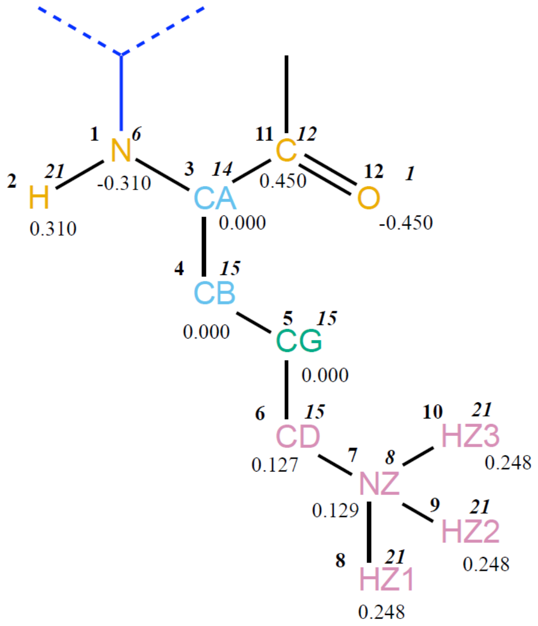

Table S1. Atoms of the ORNH building block.

Seq.	Name	IAC	Mass	Charge	Exclusions
1	N	6	14	-0.31000	2 3 4 11
2	H	21	1	0.31000	3
3	CA	14	3	0.00000	4 5 11 12 13
4	CB	15	4	0.00000	5 6 11
5	CG	15	4	0.00000	6 7
6	CD	15	4	0.12700	7 8 9 10
7	NZ	8	14	0.12900	8 9 10
8	HZ1	21	1	0.24800	9 10
9	HZ2	21	1	0.24800	10
10	HZ3	21	1	0.24800	
11	C	12	12	0.45000	
12	O	1	16	-0.45000	

Table S2. Bond types of the ORNH building block.

I	J	TYPE
1	2	2
1	3	21
3	4	27
3	11	27
4	5	27
5	6	27
6	7	21
7	8	2
7	9	2
7	10	2
11	12	5
11	13	10

Table S3. Bond angle types of the ORNH building block.

I	J	K	Type
-1	1	2	32
-1	1	3	31
2	1	3	18
1	3	4	13
1	3	11	13
4	3	11	13
3	4	5	15
4	5	6	15
8	7	9	10
8	7	10	10
9	7	10	10
3	11	12	30
3	11	13	19
12	11	13	33
5	6	7	15
6	7	8	11
6	7	9	11
6	7	10	11

Table S4. Dihedral angle types of the ORNH building block.

I	J	K	L	Type
-2	-1	1	3	14

-1	1	3	11	44
-1	1	3	11	43
1	3	4	5	34
1	3	11	13	45
1	3	11	13	42
3	4	5	6	34
8	7	6	5	29
4	5	6	7	34

Table S5. Improper dihedral angle types of the ORNH building block.

I	J	K	L	Type
1	-1	3	2	1
3	1	11	4	2
11	3	13	12	1

1.2. NLEU Building Block

Figure S2. NLEU building block with atom numbering, GROMOS integer atom codes (IAC) and partial charges: $^{\text{atom number}}X^{IAC}_{\text{partial charge}}$. Color boundaries indicate different charge groups.

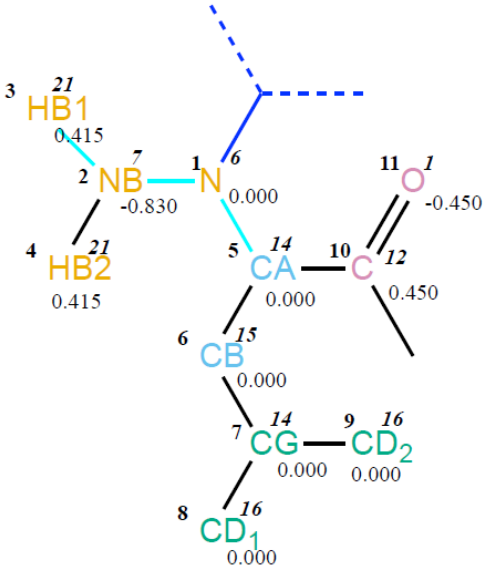

Table S6. Atoms of the NLEU building block.

Seq.	Name	IAC	Mass	Charge	Exclusions
1	N	6	14	0.00000	2 3 4 5 6 10
2	NB	7	14	-0.83000	3 4 5
3	HB1	21	1	0.41500	4
4	HB2	21	1	0.41500	
5	CA	14	3	0.00000	6 7 10 11 12
6	CB	15	4	0.00000	7 8 9 10
7	CG	14	3	0.00000	8 9
8	CD1	16	5	0.00000	9
9	CD2	16	5	0.00000	
10	C	12	12	0.45000	
11	O	1	16	-0.45000	

Table S7. Bond types of the NLEU building block.

I	J	TYPE
1	2	17
1	5	21
5	6	27
5	10	27
6	7	27
7	8	27
7	9	27
10	11	5
10	12	10
2	3	2
2	4	2

Table S8. Bond angle types of the NLEU building block.

I	J	K	Type
-1	1	2	32
-1	1	5	31
2	1	5	23
1	2	3	11
1	2	4	11
3	2	4	10
1	5	6	13
1	5	10	13
6	5	10	13
5	6	7	15
6	7	8	15
6	7	9	15

8	7	9	15
5	10	11	30
5	10	12	19
11	10	12	33

Table S9. Dihedral angle types of the NLEU building block.

I	J	K	L	Type
-2	-1	1	5	14
-1	1	5	10	44
-1	1	5	10	43
1	5	6	7	34
1	5	10	12	45
1	5	10	12	42
5	6	7	8	34
5	1	2	3	14

Table S10. Improper dihedral angle types of the NLEU building block.

I	J	K	L	Type
1	-1	5	2	1
5	1	10	6	2
6	8	9	7	2
10	5	12	11	1

GROMOS molecular topology building blocks for ORNH and for all the N-aminated amino acid residues used in this study have also been uploaded as an additional Supporting Information file.

2. NOE atom-atom distance upper bounds

Lists of NOE atom-atom distance upper bounds (in nm) derived from NMR experiments and r^{-6} averaged distances (in nm) calculated from the PepF MD simulations.

The NOE data are taken from Sarnowski et al. (Angew. Chem. Int. Ed. 2017, 56 2083-2086) unless otherwise stated. The upper distance bounds used for NOE's identified as strong, medium and weak were 0.28 nm, 0.35 nm and 0.5 nm respectively with the pseudoatom corrections of Wüthrich et al. (J. Mol. Biol. 1983 169,949-961) applied.

Table S11. PepC simulation

NOEs 1-19 from Sarnowski et. al. Angew. Chem. Int. Ed. 2017, 56 2083-2086
NOEs 20-36 from Syud et al. J. Amer. Chem. Soc. 1999 121, 11577-11578

NOE number	Residue and atom 1			Residue and atom 2			Upper bound	Distance in PepC simulation
1	1	ARG	HD@	11	LEU	HD@	0.890	0.957
2	2	TYR	HA	11	LEU	HA	0.350	0.271
3	2	TYR	HD@	9	LYSH	HB@	0.580	0.439
4	2	TYR	HD@	9	LYSH	HG@	0.580	0.514
5	2	TYR	HD@	9	LYSH	HD@	0.650	0.470
6	2	TYR	HD@	10	ILE	HA	0.550	0.564
7	2	TYR	HD@	11	LEU	HA	0.550	0.410
8	2	TYR	HD@	11	LEU	HD@	0.990	0.502
9	2	TYR	HE@	9	LYSH	HG@	0.580	0.646
10	2	TYR	HE@	9	LYSH	HD@	0.800	0.562
11	2	TYR	HE@	10	ILE	HA	0.700	0.594
12	2	TYR	HE@	11	LEU	HA	0.550	0.491
13	2	TYR	HE@	11	LEU	HD@	0.840	0.423
14	4	GLU	HA	9	LYSH	HA	0.280	0.261
15	5	VAL	HA	6	DPRO	HD@	0.380	0.216
16	5	VAL	HG@	6	DPRO	HA	0.790	0.567
17	5	VAL	HG@	6	DPRO	HD@	0.890	0.451
18	11	LEU	HD@	14	GLY	HA@	0.740	0.545
19	12	GLN	HA	13	DPRO	HD@	0.380	0.217
20	1	ARG	HN	12	GLN	HN	0.280	0.361
21	1	ARG	HN	14	GLY	HN	0.280	0.26
22	1	ARG	HB@	14	GLY	HN	0.600	0.502
23	2	TYR	HD@	9	LYSH	HA	0.700	0.585
24	2	TYR	HD@	9	LYSH	HE@	0.800	0.506
25	2	TYR	HD@	11	LEU	HB@	0.800	0.541
26	2	TYR	HE@	11	LEU	HB@	0.650	0.475
27	2	TYR	HB@	9	LYSH	HB@	0.550	0.389

28	2	TYR	HB@	9	LYSH	HG@	0.700	0.423
29	2	TYR	HB@	9	LYSH	HD@	0.700	0.376
30	5	VAL	HN	8	ORNH	HN	0.280	0.390
31	5	VAL	HG@	6	DPRO	HB@	0.890	0.718
32	6	DPRO	HA	8	ORNH	HN	0.500	0.37
33	6	DPRO	HG@	7	GLY	HN	0.600	0.472
34	6	DPRO	HD@	7	GLY	HN	0.600	0.451
35	7	GLY	HN	8	ORNH	HN	0.500	0.279
36	12	GLN	HB@	13	DPRO	HB@	0.700	0.684

Table S12. PepF simulation

NOEs 1-8 from Sarnowski Angew. Chem. Int. Ed. 2017, 56 2083-2086
NOEs 9-21 from Stanger and Gellman J. Amer. Chem. Soc. 1998 120, 4236-4237

NOE number	Residue and atom 1			Residue and atom 2			Upper bound	Distance in PepF simulation
1	2	TYR	HD@	9	LYSH	HB@	0.800	0.431
2	2	TYR	HD@	9	LYSH	HG@	0.800	0.490
3	2	TYR	HD@	11	LEU	HA	0.550	0.427
4	2	TYR	HE@	11	LEU	HA	0.550	0.515
5	2	TYR	HE@	11	LEU	HG	0.480	0.429
6	2	TYR	HE@	11	LEU	HD@	0.770	0.463
7	5	VAL	HA	6	DPRO	HD@	0.380	0.216
8	5	VAL	HG@	6	DPRO	HD@	0.670	0.450
9	2	TYR	HA	12	GLN	HN	0.500	0.314
10	2	TYR	HA	11	LEU	HA	0.500	0.279
11	11	LEU	HA	2	TYR	HE@	0.700	0.515
12	3	VAL	HN	10	ILE	HN	0.500	0.330
13	4	GLU	HA	9	LYSH	HA	0.500	0.263
14	5	VAL	HN	8	ORNH	HN	0.500	0.391
15	7	GLY	HN	8	ORNH	HN	0.500	0.282
16	2	TYR	HD@	9	LYSH	HD@	0.800	0.462
17	2	TYR	HD@	11	LEU	HD@	0.990	0.532
18	2	TYR	HE@	11	LEU	HB@	0.800	0.560
19	2	TYR	HD@	10	ILE	HA	0.700	0.573
20	10	ILE	HD@	12	GLN	HG@	0.750	0.544
21	12	GLN	HE21	10	ILE	HG2@	0.650	0.717

Table S13. PepF_N2 simulation

NOE number	Residue and atom 1			Residue and atom 2			Upper bound	Distance in PepF_N2 simulation
1	2	NTYR	HA	11	LEU	HA	0.280	0.291
2	2	NTYR	HD@	9	LYSH	HB@	0.800	0.427
3	2	NTYR	HD@	9	LYSH	HG@	0.800	0.499
4	2	NTYR	HD@	9	LYSH	HD@	0.800	0.465
5	2	NTYR	HD@	10	ILE	HA	0.480	0.563
6	2	NTYR	HD@	11	LEU	HA	0.550	0.411
7	2	NTYR	HD@	11	LEU	HD@	0.990	0.539
8	2	NTYR	HE@	9	LYSH	HD@	0.580	0.531
9	2	NTYR	HE@	9	LYSH	HE@	0.800	0.551
10	2	NTYR	HE@	11	LEU	HA	0.700	0.487
11	2	NTYR	HE@	11	LEU	HD@	0.840	0.448

12	3	VAL	HG@	12	GLN	HG@	0.670	0.441
13	4	GLU	HA	9	LYSH	HA	0.280	0.263
14	5	VAL	HA	6	DPRO	HD@	0.380	0.216
15	5	VAL	HG@	6	DPRO	HD@	0.740	0.449

Table S14. PepF_N9 simulation

NOE number	Residue and atom 1			Residue and atom 2			Upper bound	Distance in PepF_N9 simulation
1	2	TYR	HA	11	LEU	HA	0.280	0.277
2	2	TYR	HD@	9	NKH	HB@	0.650	0.434
3	2	TYR	HD@	9	NKH	HG@	0.650	0.491
4	2	TYR	HD@	9	NKH	HD@	0.800	0.474
5	2	TYR	HD@	10	ILE	HA	0.700	0.577
6	2	TYR	HD@	11	LEU	HA	0.480	0.427
7	2	TYR	HE@	9	NKH	HD@	0.580	0.486
8	2	TYR	HE@	10	ILE	HA	0.700	0.609
9	2	TYR	HE@	11	LEU	HA	0.700	0.523
10	2	TYR	HE@	11	LEU	HD@	0.840	0.476
11	3	VAL	HG@	12	GLN	HG@	0.740	0.422
12	5	VAL	HA	6	DPRO	HD@	0.380	0.216
13	5	VAL	HG@	6	DPRO	HA	0.790	0.578
14	5	VAL	HG@	6	DPRO	HD@	0.890	0.457

Table S15. PepF_N11 simulation

NOE number	Residue and atom 1			Residue and atom 2			Upper bound	Distance in PepF_N11 simulation
1	2	TYR	HA	11	NLEU	HA	0.280	0.285
2	2	TYR	HD@	9	LYSH	HB@	0.650	0.450
3	2	TYR	HD@	9	LYSH	HG@	0.650	0.507
4	2	TYR	HE@	9	LYSH	HB@	0.580	0.463
5	2	TYR	HE@	11	NLEU	HD@	0.770	0.474
6	3	VAL	HG@	12	GLN	HG@	0.890	0.462
7	5	VAL	HA	6	DPRO	HD@	0.380	0.215
8	5	VAL	HG@	6	DPRO	HA	0.640	0.568
9	5	VAL	HG@	6	DPRO	HD@	0.740	0.447
10	4	GLU	HA	9	LYSH	HA	0.280	0.272
11	4	GLU	HG@	9	LYSH	HA	0.450	0.385

Table S16. PepF_N9_N11 simulation

NOE number	Residue and atom 1			Residue and atom 2			Upper bound	Distance in PepF_N9_N11 simulation
1	2	TYR	HA	11	NLEU	HA	0.280	0.281
2	2	TYR	HA	11	NLEU	HD@	0.790	0.487
3	2	TYR	HD@	9	NKH	HD@	0.650	0.477
4	2	TYR	HD@	9	NKH	HG@	0.800	0.514
5	2	TYR	HD@	11	NLEU	HA	0.550	0.432
6	2	TYR	HE@	9	NKH	HD@	0.650	0.457
7	2	TYR	HE@	9	NKH	HG@	0.800	0.522
8	2	TYR	HE@	11	NLEU	HA	0.550	0.528
9	3	VAL	HG@	12	GLN	HA	0.640	0.600
10	3	VAL	HG@	12	GLN	HG@	0.670	0.472
11	5	VAL	HA	6	DPRO	HD@	0.380	0.217
12	5	VAL	HG@	6	DPRO	HA	0.790	0.577
13	5	VAL	HG@	6	DPRO	HD@	0.670	0.457

3 Conformational clustering analysis

Table S17. Clustering of the PepF trajectory. Populations of clusters and of the respective hydrogen bonds (%).

The clustering was performed with a cut-off RMSD of 0.1 nm for the backbone atoms of residues 2-11.

The hydrogen bonds were identified using the definition for medium-strong hydrogen bonds: H-acceptor distance < 0.25 nm and donor-H-acceptor angle > 135°. Populations < 5% are excluded.

	Cluster 1	Cluster 2	Cluster 3
Population of clusters	98.5	0.7	0.3

Hydrogen bond	Cluster 1	Cluster 2	Cluster 3
1 NH1 – 12 O	12.0	15.9	
1 NH2 – 12 O	11.8	6.1	
1 NH3 – 12 O	11.9	9.1	
1 HH11 – 4 OE1			6.3
1 HH21 – 12 OE1	7.6	6.8	
1 HH11 – 12 OE1		5.3	
1 HE – 12 OE1	5.5	7.6	
3 NH – 10 O	95.6	94.7	9.4
3 NH – 12 OE1			60.9
4 NH – 4 OE1	6.7	21.2	
4 NH – 4OE2	7.3	25.0	
5 NH – 3 O	9.3	19.7	
5 NH – 4 OE1		5.3	
5 NH – 4 OE2		5.3	
5 NH – 8 O	74.7	11.4	93.8
8 NH – 5 O	36.0	7.6	34.4
8 NH – 6 O	16.1		10.9
9 NH – 7 O		37.9	
9 HNZ* – 6 O		7.6	
10 NH – 3 O	81.5	25.8	87.5
10 NH – 8 O		14.4	
12 NH – 1 O	76.0	84.1	6.2
12 NH – 10 O			25.0
12 HE21 – 1 O			15.6
12 HE21 – 4 OE1		9.9	
12 HE21 – 4 OE2		6.1	

* The highest hydrogen bond population from the donor NZ-HZ1, NZ-HZ2 or NZ-HZS3 is listed

Table S18. Clustering of the PepE trajectory. Populations of clusters and of the respective hydrogen bonds (%).

The clustering was performed with a cut-off RMSD of 0.1 nm for the backbone atoms of residues 2-11.

The hydrogen bonds were identified using the definition for medium-strong hydrogen bonds: H-acceptor distance < 0.25 nm and donor-H-acceptor angle > 135°. Populations < 5% are excluded.

	Cluster 1	Cluster 2	Cluster 3	Cluster 4
Population of clusters	54.6	6.2	4.8	3.7

Hydrogen bond	Cluster 1	Cluster 2	Cluster 3	Cluster 4
1 HNE – 3 O			6.3	
1 HNE – 6 O			6.8	
1 HNE – 10 O		5.8		
1 HNE – 12 O				24.7
1 HH11 – 4 O			7.7	
1 HH21 – 6 O			32.6	
1 HH21 – 9 O				11.4
1 HH 11 – 12 O				10.3
2 NH – 10 O			19.1	
2 NH – 11 O		30.5		
2 NH – 12 O			62.0	
2 NH – 12 OE1				61.0
3 NH – 11 O	41.7			
3 NH – 12 OE1	12.0			
3 NH – 12 O	5.3		74.4	
4 NH – 7 O				80.4
4 HN – 4 OE1		35.4	25.1	
4 HN – 4 OE2		32.8	30.5	
5 NH – 9 O	89.1			
5 NH – 4 OE1		25.4	15.7	44.7
5 NH – 4 OE2		23.1	13.7	17.4
7 NH – 12 OE1			25.3	
8 NH – 4 OE1	12.2			
8 NH – 4 OE2	12.1			
8 NH – 6 O	11.0			
8 NH – 12 OE1		13.1	7.7	
9 NH – 2 O				95.6
9 HN – 7 O	63.4		22.3	
9 NH – 12 OE1			66.8	
11 NH – 3 O	90.8			
12 NH – 3 O		5.9	72.2	
12 NH – 9 O			64.2	18.5
12 NH – 10 O			11.0	21.7
12 NH – 12 OE1	12.6			

12 HE22 – 2 OH		6.0		
12 NH2* – 5 O			61.0	
12 NH2* - 9 O			10.7	

* C-terminal NH_2 group

Table S19. Combined clustering of the PepF and PepE trajectories. Populations of clusters and of the respective hydrogen bonds (%).

The clustering was performed with a cut-off RMSD of 0.1 nm for the backbone atoms of residues 2-11.

The hydrogen bonds were identified using the definition for medium-strong hydrogen bonds: H-acceptor distance < 0.25 nm and donor-H-Acceptor angle > 135°. Populations < 5% are excluded.

	Cluster 1	Cluster 2	Cluster 3
Population of clusters by PepF	98.3	0	0
Population of clusters by PepE	0	54.6	6.1

Hydrogen bond	Cluster 1	Cluster 2	Cluster 3
1 NH1 – 12 O	12.0		
1 NH2 – 12 O	12.0		
1 NH3 – 12 O	11.8		
1 HNE – 10 O			6.4
1 HNE – 12 OE1	5.6		
1 HH21 – 12 OE1	7.4		
2 NH – 11 O			29.7
2 HN – 12 O			50.0
3 NH – 10 O	96.0		
3 NH – 11 O		41.3	
3 NH – 12 O		5.5	93.6
3 NH – 12 OE1		12.3	
4 HN – 4 OE1	6.6		31.2
4 HN – 4 OE2	7.4		34.8
5 NH – 3 O	9.5		
5 NH – 4 OE1			25.7
5 NH – 4 OE2			23.1
5 NH – 8 O	74.6		
5 NH – 9 O		89.1	
8 NH – 4 OE1		12.3	
8 NH – 4 OE2		12.7	
8 NH – 5 O	36.3		
8 NH – 6 O	15.8	10.7	
8 NH – 12 OE1			13.9
9 NH – 7 O		64.2	
9 NH – 5 O		10.7	
9 NH – 12 OE1			54.9
10 NH – 3 O	81.5		
10 NH – 8 O		7.1	
11 NH – 3 O		98.4	
12 NH – 1 O	76.5		
12 NH – 9 O			40.9
12 NH – 12 OE1		12.6	

12 HE22 – 2 OH			5.1
12 NH2* – 1 O		8.2	
12 NH2* - 3 O			6.3
12 NH2* – 9 O			55.9

* C-terminal NH_2 group

Table S20. Clustering of the PepF_N9_N11 trajectory. Populations of clusters and of the respective hydrogen bonds (%).

The clustering was performed with a cut-off RMSD of 0.1 nm for the backbone atoms of residues 2-11.

The hydrogen bonds were identified using the definition for medium-strong hydrogen bonds: H-acceptor distance < 0.25 nm and donor-H-Acceptor angle > 135°. Populations < 5% are excluded.

	Cluster 1	Cluster 2	Cluster 3
Population of clusters	99.0	0.5	0.3

Hydrogen bond	Cluster 1	Cluster 2	Cluster 3
1 NH2 – 11 O		5.0	
1 NH1 – 12 O	13.7	18.0	5.7
1 NH2 – 12 O	13.6	9.0	10.0
1 NH3 – 12 O	13.4	12.0	5.7
1 NH1 – 12 OE1			8.6
1 NH2 – 12 OE1			11.4
1 NH3 – 12 OE1			8.6
1 HH21 – 12 OE1	6.4		
2 OH – 4 OE1		23.0	
2 OH – 4 OE2		16.0	
3 NH – 1 O			18.6
3 NH – 10 O	93.0	95.0	42.9
4 NH – 4 OE1		8.0	
4 NH – 4 OE2		11.0	
5 NH – 3 O			17.2
5 NH – 4 OE1		8.0	
5 NH – 4 OE2		14.0	
5 NH – 8 O	81.8	55.0	61.4
5 NH – 12 OE1			
7 NH – 4 O			10.0
8 NH – 5 O	48.6	40.0	22.9
8 NH – 6 O	10.0		45.7
9 HNB1 – 7 O	6.6	42.0	
9 HNB2 – 9 O	8.3	8.0	
9 HZ1 – 6 O		8.0	
9 HZ2 – 6 O		6.0	
10 NH – 3 O	85.4	68.0	61.4
10 NH – 8 O		5.0	
11 HNB1 – 9 O		15.0	
12 NH – 1 O	61.8	51.0	32.9
12 NH – 10 O			11.4

Table S21. Clustering of the PepE_N9_N11 trajectory. Populations of clusters and of the respective hydrogen bonds (%).

The clustering was performed with a cut-off RMSD of 0.1 nm for the backbone atoms of residues 2-11.

The hydrogen bonds were identified using the definition for medium-strong hydrogen bonds: H-acceptor distance < 0.25 nm and donor-H-acceptor angle > 135°. Populations < 5% are excluded.

	Cluster 1	Cluster 2	Cluster 3
Population of clusters	21.8	17.7	10.3

Hydrogen bond	Cluster 1	Cluster 2	Cluster 3
1 NH1 – 10 O	19.9		
1 NH2 – 10 O	20.5		
1 NH3 – 10 O	20.2		
1 NH1 – 12 O		5.3	
1 NH3 – 12 O		5.7	
1 NH1 – 12 OE1	6.6		
1 NH2 – 12 OE1	6.7		
1 NH3 – 12 OE1	6.1		
1 HNE – 12 O			10.9
1 HH11 – 12 O			7.0
1 HH21 – 12 O			15.0
2 NH – 11 O		9.1	59.5
2 NH – 12 OE1			5.2
2 OH – 4 OE1		11.9	
2 OH – 4 OE2		11.7	
3 NH – 8 O	96.1		
3 NH – 10 O		78.1	
4 NH – 4 OE1	32.6		14.1
4 NH – 4 OE2	31.5		13.8
5 NH – 3 O			27.7
5 NH – 4 OE1	11.0		
5 NH – 4 OE2	10.1		
5 NH – 8 O		85.7	
5 NH – 12 OE1			14.7
7 NH – 4 O	74.9		
8 NH – 3 O	67.0		
8 NH – 5 O	8.2	50.4	
9 HNB1 – 7 O		8.9	24.6
9 HNB2 – 9 O	7.9	9.6	15.3
9 HNB2 – 12 O			17.2
9 HNB1 – 12 OE1			12.5
9 HNB2 – 12 OE1			10.8
10 NH – 1 O	61.2		
10 NH – 3 O		87.6	

11 HNB1 – 9 O	14.2		
12 NH – 2 O			69.9
12 NH – 9 O			30.9
12 NH – 10 O	7.1		15.9
12 HE21 – 7 O			9.5
12 HE21 – 9 O	8.6		
12 HE22 – 3 O			6.1
12 NH – 12 OE1	5.6	5.7	
12 NH2* – 3 O			6.8
12 NH2* – 4 OE1			10.3
12 NH2* – 4 OE2			12.6

* C-terminal NH_2 group

Table S22. Combined clustering of the PepF_N9_N11 and PepE_N9_N11 trajectories. Populations of clusters and of the respective hydrogen bonds (%).

The clustering was performed with a cut-off RMSD of 0.1 nm for the backbone atoms of residues 2-11.

The hydrogen bonds were identified using the definition for medium-strong hydrogen bonds: H-acceptor distance < 0.25 nm and donor-H-acceptor angle > 135°. Populations < 5% are excluded.

	Cluster 1	Cluster 2	Cluster 3
Population of clusters by PepF_N9_N11	98.7	0	0
Population of clusters by PepE_N9_N11	16.3	21.8	10.1

Hydrogen bond	Cluster 1	Cluster 2	Cluster 3
1 NH1 – 10 O		19.9	
1 NH2 – 10 O		20.5	
1 NH3 – 10 O		19.6	
1 NH1 – 12 O	12.5		
1 NH2 – 12 O	12.5		
1 NH3 – 12 O	12.4		
1 NH1 – 12 OE1		6.3	
1 NH2 – 12 OE1		6.7	
1 NH3 – 12 OE1		5.7	
1 HNE – 12 O			11.3
1 HH11 – 12 O			7.1
1 HH21 – 12 O			16.0
1 HH21 – 12 OE1	5.5		
2 NH – 11 O			63.7
3 NH – 8 O		96.0	
3 NH – 10 O	91.6		
4 NH – 4 OE1		31.8	13.6
4 NH – 4 OE2		31.4	13.0
5 NH – 3 O			30.5
5 NH – 4 OE1		11.6	
5 NH – 4 OE2		10.7	
5 NH – 8 O	82.5		
5 NH – 12 OE1			13.2
7 NH – 4 O		75.0	
8 NH – 3 O		68.1	
8 NH – 5 O	49.6	7.2	
8 NH – 6 O	9.1		
9 HNB1 – 7 O	7.1		23.4
9 HNB2 – 9 O	8.6	7.9	17.4
9 HNB2 – 12 O			16.0

9 HNB1 – 12 OE1			13.7
9 HNB2 – 12 OE1			10.3
9 HZ3 – 5 O			5.1
10 NH – 1 O		60.3	
10 NH – 3 O	86.0		
11 HNB1 – 9 O		14.7	
12 NH – 1 O	52.9		
12 NH – 2 O			66.3
12 NH – 9 O			28.4
12 NH – 10 O		7.3	14.9
12 HE21 – 7 O			9.5
12 HE21 – 9 O		8.5	
12 HE22 – 3 O			6.0
12 NH – 12 OE1		5.8	
12 NH2* – 3 O			7.1
12 NH2* – 4 OE1			8.4
12 NH2* – 4 OE2			11.6

* C-terminal NH_2 group

Table S23. Clustering of the PepF_N2_N4_N9_N11 trajectory. Populations of clusters and of the respective hydrogen bonds (%).

The clustering was performed with a cut-off RMSD of 0.1 nm for the backbone atoms of residues 2-11.

The hydrogen bonds were identified using the definition for medium-strong hydrogen bonds: H-acceptor distance < 0.25 nm and donor-H-Acceptor angle > 135°. Populations < 5% are excluded.

	Cluster 1	Cluster 2	Cluster 3
Population of clusters	97.6	2.0	0.3

Hydrogen bond	Cluster 1	Cluster 2	Cluster 3
1 NH1 – 12 O	20.9	10.6	7.3
1 NH2 – 12 O	18.3	16.0	18.2
1 NH3 – 12 O	19.8	12.0	18.2
1 NH2 – 12 OE1		5.9	
1 HH21 – 12 OE1	10.2	5.2	12.7
1 HE – 12 OE1		10.3	10.9
2 OH – 4 OE1	8.6	14.3	
2 OH – 4 OE2	7.5	9.1	
3 NH – 10 O	94.7	88.7	89.1
4 HNB1 – 2 O	9.2	19.9	
5 NH – 3 O			14.6
5 NH – 8 O	75.5	20.2	63.6
7 NH – 4 O			5.5
7 NH – 4 OE1			5.5
8 NH – 5 O	57.2	26.0	36.4
8 NH – 6 O	5.7		43.6
9 HNB1 – 7 O	13.8	52.1	
9 HNB2 – 9 O	8.5	10.1	
9 HNZ* – 4 O		12.8	
9 HNZ* – 6 O		7.7	
9 HNZ* – 7 O			5.5
10 NH – 3 O	86.4	69.8	81.8
11 HNB1 – 9 O	8.3	15.0	
12 NH – 1 O	67.9	40.3	70.9

* The highest hydrogen bond population from the donor NZ-HZ1, NZ-HZ2 or NZ-HZS3 is listed

Table S24. Clustering of the PepE_N2_N4_N9_N11 trajectory. Populations of clusters and of the respective hydrogen bonds (%).

The clustering was performed with a cut-off RMSD of 0.1 nm for the backbone atoms of residues 2-11.

The hydrogen bonds were identified using the definition for medium-strong hydrogen bonds: H-acceptor distance < 0.25 nm and donor-H-Acceptor angle > 135°. Populations < 5% are excluded.

	Cluster 1	Cluster 2	Cluster 3	Cluster 4
Population of clusters	33.7	5.9	3.3	3.1

Hydrogen bond	Cluster 1	Cluster 2	Cluster 3	Cluster 4
1 NH1 – 12 O	15.2		20.0	
1 NH2 – 12 O	14.2		12.7	
1 NH3 – 12 O	15.0		14.2	
1 HH21 – 12 O				5.5
1 HH21 – 12 OE1	6.8			
2 HNB2 – 9 O		43.6		
2 HNB2 – 11 O	5.3		4.6	
2 OH – 4 OE1	7.3	36.5	10.9	
2 OH – 4 OE2	16.8	37.8	10.5	7.4
3 NH – 10 O	92.5		92.4	
4 HNB1 – 2 O	9.6	67.6	18.8	22.4
4 HNB2 – 8 O		94.4		
5 NH – 8 O	76.1		12.0	
5 NH – 4 OE1				41.2
5 NH – 4 OE2				28.9
7 NH – 4 O		60.5		
8 NH – 4 OE1		43.2		
8 NH – 5 O	56.7		24.7	
8 HNZ* – 2 O				15.5
9 HNB1 – 7 O	15.7	6.1	39.9	
9 HNB2 – 9 O	8.6		8.6	
9 HNZ* – 4 O			9.4	
9 HNZ* – 6 O			5.9	
10 NH – 3 O	84.1		65.9	
11 HNB1 – 9 O	6.4		14.2	5.3
12 NH – 1 O	47.9		32.6	

* The highest hydrogen bond population from the donor NZ-HZ1, NZ-HZ2 or NZ-HZS3 is listed

Table S25. Combined clustering of the PepF_N2_N4_N9_N11 and PepE_N2_N4_N9_N11 trajectories. Populations of clusters and of the respective hydrogen bonds (%).

The clustering was performed with a cut-off RMSD of 0.1 nm for the backbone atoms of residues 2-11.

The hydrogen bonds were identified using the definition for medium-strong hydrogen bonds: H-acceptor distance < 0.25 nm and donor-H-acceptor angle > 135°. Populations < 5% are excluded.

	Cluster 1	Cluster 2	Cluster 3
Population of clusters by PepF_N2_N4_N9_N11	97.6	0	1.9
Population of clusters by PepE_N2_N4_N9_N11	33.7	5.9	3.3

Hydrogen bond	Cluster 1	Cluster 2	Cluster 3
1 NH1 – 12 O	19.6		16.3
1 NH2 – 12 O	18.6		13.8
1 NH3 – 12 O	17.3		14.1
1 HH21 – 12 OE1	9.3		
2 OH – 4 OE1	8.3	36.5	12.1
2 OH – 4 OE2	9.8	37.5	10.2
2 HNB2 – 9 O		47.4	
3 NH – 10 O	94.3		92.3
4 HNB1 – 2 O	9.4	67.6	18.8
4 HNB2 – 8 O		95.4	
5 NH – 8 O	76.3		14.6
7 NH – 4 O		61.0	
8 NH – 4 OE1		43.8	
8 NH – 4 OE2		42.8	
8 NH – 5 O	57.3		26.1
8 NH – 6 O	5.4		
9 HNB1 – 7 O	14.7	6.1	44.2
9 HNB2 – 9 O	8.4		9.4
9 HNZ* – 4 O			9.8
9 HNZ* – 6 O			5.8
10 NH – 3 O	85.7		68.6
11 HNB1 – 9 O	7.7		13.4
12 NH – 1 O	62.6		36.4
12 NH – 12 OE1		5.1	

* The highest hydrogen bond population from the donor NZ-HZ1, NZ-HZ2 or NZ-HZS3 is listed
